# AgamOBP1‐Directed Discovery of Repellents to Control the Spread of Mosquito‐Borne Diseases

**DOI:** 10.1002/cmdc.202500555

**Published:** 2025-09-21

**Authors:** Evanthia Chazapi, Eftichia Kritsi, Constantinos Potamitis, Panagiota G. V. Liggri, Katerina E. Tsitsanou, Christina E. Drakou, Antonios Michaelakis, Dimitrios P. Papachristos, Spyros E. Zographos, Maria Zervou, Theodora Calogeropoulou

**Affiliations:** ^1^ Institute of Chemical Biology National Hellenic Research Foundation 48 Vassileos Constantinou Ave. 11635 Athens Greece; ^2^ Benaki Phytopathological Institute Scientific Directorate of Entomology and Agricultural Zoology 8 Stefanou Delta Str., Kifissia 14561 Athens Greece; ^3^ Present address: Laboratory of Chemistry, Analysis & Design of Food Processes, Department of Food Science and Technology University of West Attica Agiou Spyridonos 12243 Egaleo Greece; ^4^ Present address: Cloudpharm Private Company Zosimadon 13, Aigaleo 12243 Athens Greece; ^5^ Present address: Department of Biochemistry and Biotechnology University of Thessaly Biopolis 41500 Larissa Greece

**Keywords:** ^1^H STD NMR spectroscopy, amines, *Anopheles gambiae* odorant binding protein 1, dynamic combinatorial chemistry, mosquito repellent

## Abstract

Toward the discovery of novel efficient repellents, protein‐directed dynamic combinatorial chemistry (pdDCC) coupled to saturation‐transfer difference (STD) NMR spectroscopy was initially employed to identify modulators of the malaria vector *Anopheles gambiae* Odorant Binding Protein 1 (AgamOBP1). A library of potential binders of AgamOBP1 (secondary amines) generated from two amines and seven aldehydes was designed aiming to enable interactions with critical amino acids at the DEET‐site and to bridge the DEET‐ and Icaridin sIC‐binding pockets, both implicated in repellents recognition. Solubility issues hindered the clear identification of binders among the DCL members, except for one sublibrary, leading us to shift our strategy towards the synthesis of the designed amines, followed by direct evaluation of their binding to AgamOBP1 using ^1^H STD NMR spectroscopy. The identified binders were further validated in vitro by fluorescence competition assays, and the most potent compounds which also possessed suitable vapor pressure were evaluated as repellents in arm‐in‐cage behavioral assays against *Aedes albopictus*. Amines **2A**, **3A**, **4A**, and **6A** showed significant repellent activity. The most potent was compound **4A** (4‐methyl‐*N*‐(pyridin‐4‐ylmethyl)aniline) which acted as a a DEET‐like repellent at 0.4 μL cm^−^^2^ dose. Thus, our strategy showcased a promising scaffold for further optimization toward efficient mosquito repellents.

## Graphical abstract

Toward the discovery of novel mosquito repellents, a library of potential binders of AgamOBP1 (secondary amines) generated from two amines and seven aldehydes was designed aiming to enable interactions with critical amino acids at the DEET‐site and to bridge the DEET‐ and Icaridin sIC‐binding pockets. ^1^H STD NMR spectroscopy in the presence of AgamOBP1 enabled the identification of binders. The behavioral bioassay assessment of repellent activity highlighted **4A** as a DEET‐like repellent at 0.4 μL cm^‐^^2^ dose.

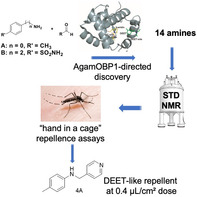

## 1 Introduction

Vector‐borne diseases (VBD) account for more than 17% of all infectious diseases, causing more than 700,000 deaths annually (WHO, 2020).^[^ ^1^ ^]^ In particular, mosquito‐borne diseases represent an important public health challenge, with millions of cases reported annually across diverse geographical regions. They are transmitted to humans and animals through hematophagous female mosquitoes, infected with parasites and viruses, belonging primarily to the *Anopheles*, *Aedes*, and *Culex* species.^[^ ^2^ ^]^ The most important mosquito‐borne diseases are malaria, dengue fever, chikungunya, Zika virus, West Nile virus, and yellow fever. Malaria, caused by parasites of the genus *Plasmodium*—particularly *Plasmodium falciparum* and *Plasmodium vivax*—remains one of the most prominent mosquito‐borne infection, annually accounting for 249 million cases and 608,000 deaths, predominantly in tropical and subtropical regions.^[^ ^3^ ^]^ Similarly, arboviral diseases like dengue and Zika virus, transmitted by *Aedes* invasive mosquito species, have expanded geographically due to urbanization, global travel, and climate change.^[^ ^4^ ^,^ ^5^ ^]^ Public health disruptions, including wars and pandemics such as COVID‐19, further exacerbate mosquito‐borne disease transmission.^[^ ^6^ ^,^ ^7^ ^]^

Prevention and control strategies rely on integrated vector management, which includes biocide applications and source reduction through the elimination of mosquito breeding sites.^[^ ^8^ ^]^ However, the increasing resistance of mosquito vectors to commonly used insecticides, such as pyrethroids, poses a significant challenge to current control programs.^[^ ^9^ ^,^ ^10^ ^]^ This highlights the urgent need for novel tools to mitigate the impact of mosquito‐borne diseases worldwide. In addition, personal protection measures, such as the use of repellents remain an important supplementary approach for reducing human exposure to mosquito bites.^[^ ^11^ ^]^ Long‐lasting repellents can disrupt the olfactory‐driven host‐seeking behavior of mosquitoes, thereby reducing bite frequency and lowering the probability of pathogen transmission.

**Figure 1:**
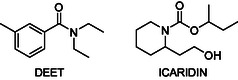
Chemical structures of the DEET and icaridin.

The most widely used insect repellents are DEET (*N,N*‐diethyl‐3‐methylbenzamide) and Icaridin also known as Picaridin (butan‐2‐yl 2‐(2‐hydroxyethyl) piperidine‐1‐carboxylate) (**Figure** **1**).^[^ ^12^ ^,^ ^13^ ^]^ DEET, a carboxamide derivative developed in 1954, has been proven effective against a variety of insects, including mosquitoes, black flies, ticks, fleas, bugs, and mites.^[^ ^14^ ^]^ Icaridin is an efficient, less irritating DEET alternative that was developed in the 1980s and is effective against mosquitoes, biting flies, and ticks.^[^ ^14^ ^,^ ^15^ ^]^

However, like all existing insect repellents, DEET and Icaridin lose their effectiveness shortly after their application. In addition, their widespread use has contributed to the development of repellent resistance or insensitivity in mosquitoes.^[^ ^16^, ^17^, ^18^, ^19^, ^20^, ^21^, ^22^, ^23^ ^–^ ^24^ ^]^ Moreover, despite the excellent long‐term safety profiles of DEET and Icaridin, concerns have been raised regarding their potential toxicity and carcinogenicity to mammals.^[^ ^25^, ^26^ ^–^ ^27^ ^]^ In addition, animal studies have shown the potential of Icaridin to cause skin irritation.^[^ ^28^ ^,^ ^29^ ^]^ Therefore, there is an urgent need to develop novel repellents with a longer duration of action, reduced toxicity, minimal effective dose, and broad‐spectrum efficacy against various mosquito vectors.

Mosquito olfactory mechanisms represent an ideal target for the rational design of new repellents. Mosquitoes rely on their olfactory system to communicate with their environment, enabling them to detect plant or animal hosts for nectar and blood‐feeding, respectively, and to select suitable oviposition sites. Volatile odorants in the air are captured by the odorant binding proteins (OBPs), abundantly expressed in the insect's sensilla lymph, and are transferred to the odorant receptors (ORs) located in the olfactory sensory neurons (OSNs) within the olfactory epithelium.^[^ ^30^ ^]^ Each OBP can recognize and bind a specific class of structurally similar odorants while ORs may respond to a broader spectrum of odorant molecules. Therefore, while neither OBPs nor ORs are highly specific, they work together as a two‐step filter, contributing to the remarkable specificity of the insect olfactory system.

Although recent advancements in cryo‐EM techniques have enhanced our understanding of OR‐ligand interactions, the current knowledge is still limited. As a result, ORs are still not considered suitable targets for the design of new insect repellents.^[^ ^31^, ^32^ ^–^ ^33^ ^]^ On the other hand, OBPs constitute a useful alternative, as there is a more detailed understanding of their structure and function.^[^ ^34^, ^35^ ^–^ ^36^ ^]^ Structural studies have shown that DEET and other repellents are transported by binding to the relevant OBP proteins.^[^ ^37^, ^38^, ^39^ ^–^ ^40^ ^]^ In particular, the African malaria vector*, Anopheles gambiae*, encodes 60 different OBPs, many of which are expressed in the female antennae in high concentrations (≈10 mM). Previous studies of the *Anopheles gambiae* OBP1 (AgamOBP1) protein revealed that its mRNA is present in the antennae of female mosquitoes at much higher concentrations than in males. Noteworthy, a significant decrease in its expression levels occurs after a blood meal, suggesting that OBP1 plays an important role in the host‐seeking behavior of mosquitoes.^[^ ^41^ ^,^ ^42^ ^]^

AgamOBP1, belonging to the classical OBP subfamily, consists of six *α*‐helices connected with flexible loops and three intramolecular disulfide bonds. The crystal structures of AgamOBP1 in complex with DEET (PDB ID: 3N7H) and Icaridin (PDB ID: 5EL2) have revealed the binding sites and interactions of these two compounds.^[^ ^37^ ^,^ ^40^ ^]^ Thus, DEET was shown to bind with high shape complementarity to a binding site located at the entrance of a long hydrophobic tunnel running through the protein monomer.^[^ ^40^ ^]^ Moreover, AgamOBP1‐Icaridin complex structure revealed that Icaridin binds not only to the DEET‐binding site but can also diffuse deeper into the tunnel to occupy a second (sIC)‐binding site located near the C‐terminal region of the protein. All the information derived from comparing the binding modes of DEET and Icaridin showcased several common structural features that could be exploited for the design of novel binders of AgamOBP1.^[^ ^37^ ^]^ AgamOBP1 exhibits high sequence and structural similarity with its homologs from various disease vectors, including the Indo‐Pakistan malaria mosquito *Anopheles stephensi* (92.6%), the West Nile virus (WNV) vector *Culex quinquefasciatus* (90.4%), the Yellow fever mosquito *Aedes aegypti* (83.2%), and the aggressive, day‐time biting Asian tiger mosquito, *Aedes albopictus*—an invasive species to Europe that transmits diseases such as chikungunya, dengue, and Zika (72.1%). Given its high conservation across species, AgamOBP1 serves as a robust protein model for in silico and in vitro discovery of novel broad‐spectrum repellents.^[^ ^43^, ^44^ ^–^ ^45^ ^]^

In this work, in order to identify small molecule modulators of AgamOBP1 we set out to employ protein‐directed dynamic combinatorial chemistry (pdDCC)^[^ ^46^, ^47^, ^48^ ^–^ ^49^ ^]^ coupled to saturation‐transfer difference (STD) spectroscopy. A similar approach has successfully identified inhibitors for aspartic protease endothiapepsin, a model enzyme used for the drug development targeting diseases as hypertension, AIDS and malaria.^[^ ^50^ ^]^ pdDCC is considered an efficient approach to identify ligands for a protein of interest. The main advantage is the fact that by utilizing a reversible reaction, the protein serves as a template to shift the thermodynamically controlled reaction to a more stable product according to Le Chatelier principle. Furthermore, STD spectroscopy allows the in situ identification of the products that bind to the protein active site since only these resonances are visible in the ^1^H STD NMR spectrum. The reversible chemistry of choice was imine formation from an aldehyde and a primary amine, while the subsequent reduction of the mixture of imines affords the more stable secondary amines. To more judiciously select the dynamic library counterparts, we applied structure‐based *de novo* design, using the previously reported crystal structures of AgamOBP1 with DEET and Icaridin. However, solubility issues encountered during the library formation resulted in low signal‐to‐noise ratio in the ^1^H STD spectra; thus, only one small dynamic library afforded usable results. Thus, we switched our strategy to a more traditional medicinal chemistry approach, synthesizing the designed analogs leading to a series of 14 secondary amine derivatives. These were studied using ^1^H STD NMR spectroscopy in the presence of AgamOBP1 to assess their binding to the protein. Amines **2A**, **3A**, **4A**, **1B**, **2B**, and **5B** were selected for the fluorescent probe displacement assay using AgamOBP1 and *N*‐phenyl‐1‐naphthylamine. Compounds **2A**, **3A**, **4A**, and **6A** showed the highest potency and were further evaluated for their repellent efficacy against *Aedes albopictus.* All the tested compounds showed significant repellent activity. In particular, compound **4A** (4‐methyl‐*N*‐(pyridin‐4‐ylmethyl)aniline) featuring a pyridine moiety acted as a DEET (*N, N*‐diethyl‐3‐methylbenzamide)‐like repellent at the high‐dose and as a middle repellent at the low‐dose showcasing a promising scaffold for further development.

## 2 Results and Discussion

### 2.1 Design and Preparation of the Dynamic Combinatorial Libraries and ^1^H STD NMR Experiments

Initially, we aimed to apply protein‐directed dynamic combinatorial chemistry (pdDCC) coupled to saturation‐transfer difference (STD) spectroscopy to identify modulators of AgamOBP1. The reversible formation of Schiff bases from aromatic amines and aromatic aldehydes was the chemistry of choice for the generation of the dynamic libraries (DCL). The intermediate imines were subsequently reduced in situ to afford the final library of the stable corresponding secondary amines. Thus, the choice of the secondary amine chemotype of the current work was imposed by the chemistry used in the pDCC.

Structure‐based *de novo* design was used to propose secondary amines, potential AgamOBP1 binders, with the following objectives: a) to mimic the aromaticity of DEET through the incorporation of aromatic rings, b) to enable interactions with critical amino acids at the DEET‐site, and c) to investigate potential bridging of DEET‐ and Icaridin sIC‐sites. Such molecules, that retain the DEET binding mode and bridge the two individual binding sites through expansion into the sIC‐site, can exploit a larger available protein space, form a higher number of functional protein–repellent interactions and consequently may exhibit enhanced binding affinity and specificity. Thus, they could be detected by AgamOBP1 at lower concentrations, while still being recognized by the odorant receptors relevant to DEET and Icaridin.^[^ ^43^ ^]^

Consequently, we implemented in silico docking studies at both DEET‐binding site defined by the *α*4, *α*5, and *α*6 helices of AgamOBP1 (PDB ID: 3N7H) and the Icaridin sIC‐binding site, located deeper into the channel which includes residues from the C‐terminal region as well as helices *α*1, *α*3, *α*4, and *α*6 (PDB id: 5EL2). Our in silico studies resulted in the selection of two primary amines as the “scaffold molecules,” namely *p*‐toluidine (**A**) and 4‐(2‐aminoethyl) benzenesulfonamide (**B**) and seven aromatic aldehydes (**1–7**) as counterparts (**Table** **1**). It should be pointed out that all the proposed amines **1A–7A** and **1B–7B** (Table 1) resulting from the reduction of the imines of the DCL, exhibited higher docking scores than DEET or Icaridin at the DEET or the sIC‐binding sites, respectively (Table S1 and Table S2, Supporting Information).

**Table 1:** Design of the dynamic libraries (DCLs).

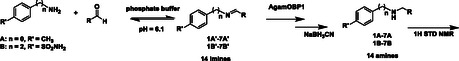				
Aldehydes **1–7**	Imines **1A′–7A′**	Amines **1A–7A**	Imines **1B′–7B′**	Amines **1B–7B**
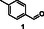	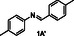	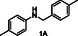	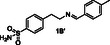	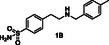
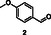	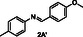	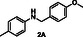	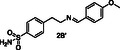	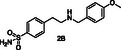
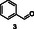	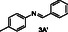	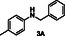	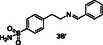	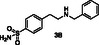
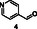	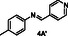	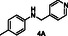	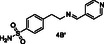	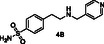
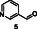	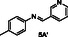	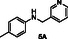	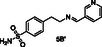	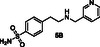
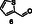	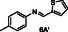	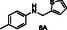	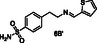	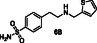
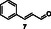	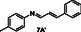	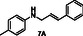	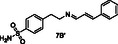	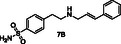

**Figure 2:**
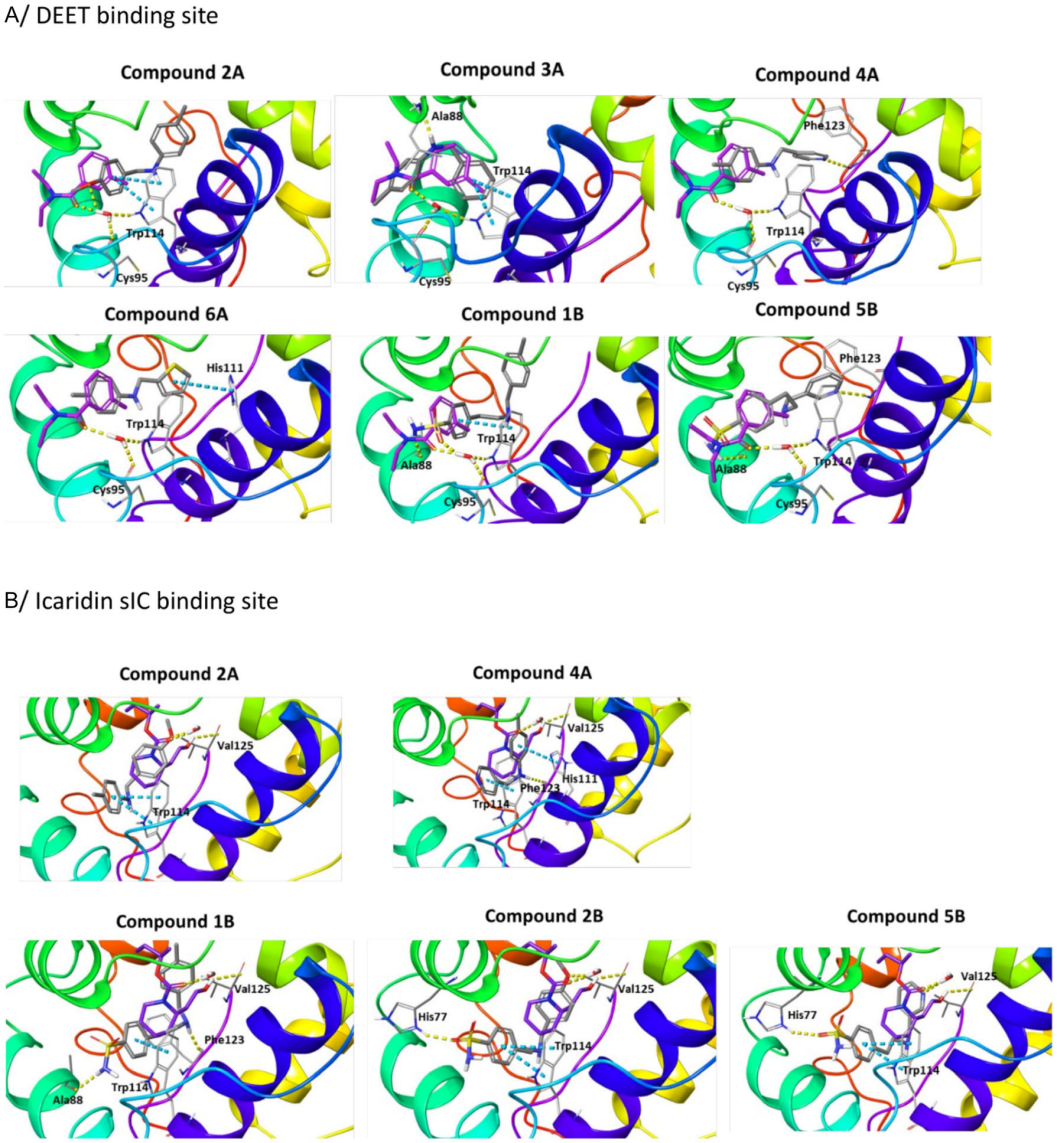
A) DEET binding site (PDB ID:3N7H). Representative binding poses of amines **2A**, **3A**, **4A**, **6A**, **1B** and **5B** (in gray) overlaid with DEET (in purple). Upon binding, DEET interacts with its carbonyl group with a conserved water molecule, which in turn forms H bonds with Trp114 (*α*6 helix) and Cys95 (loop preceding *α*5 helix); B) Icaridin sIC‐binding site (PDB ID:5EL2). Representative binding poses of the amines **2A**, **4A**, **1B**, **2B** and **5B** (in gray) overlaid with Icaridin (in purple). In this site Icaridin is involved in a direct hydrogen bond interaction with Val125 (C‐terminal amino acid) and water‐mediated hydrogen bond interactions to Met55 (*α*3 helix) and His111 (*α*6 helix).

In general, at the DEET‐site, the secondary amines based on *p*‐toluidine (A series) orient the *p*‐methylphenyl moiety towards the DEET aromatic ring with **4A** and **6A** displaying the best overlay. Notably, both the pyridine ring of **4A** and the thiophene moiety of **6A** are accommodated within the hydrophobic channel which traverses the protein and link the DEET‐ and the sIC‐binding sites via fruitful interactions. Specifically, in the case of **4A** the nitrogen of the pyridine ring interacts through H‐bond with Phe123, located at the C‐terminal loop at the sIC‐binding site. In the case of **6A**, the thiophene is involved in pi–pi interactions with His111, shown to be involved in water mediated H–bond with Icaridin bound at the sIC‐site (**Figure** **2**A). On the other hand, compound **2A**, bearing a *p*‐methoxyphenyl group, and compound **3A**, substituted by a phenyl group, target preferentially only the DEET‐site and stabilize their binding through pi–pi interactions with Trp114, a key residue previously shown to be involved in water mediated hydrogen bond interactions with DEET or Icaridin.^[^ ^37^ ^,^ ^40^ ^]^ Analog **3A** stabilizes further its binding through H‐bonding between the NH group and the backbone of Ala88 (*α*5 helix, located at the entrance of the DEET‐site). Interestingly, in the case of **2A**, the binding motif is reversed allowing the methoxy oxygen to establish a DEET‐like water bridge with Trp114 and Cys95 (Figure 2A).

Upon binding at the DEET‐site, the secondary amines based on 4‐(2‐aminoethyl) benzenesulfonamide (B series) illustrated a common pattern orienting the benzenesulfonamide toward the DEET aromatic ring (Figure 2A). The sulfonamide mimics the DEET amide with one of the two oxygens interacting with Trp114 and Cys95 via the conserved water (as seen in **1B**), whereas the binding is further stabilized through pi–pi interaction with Trp114 (see **1B**) and a direct H–bond interaction between Ala88 and the sulfonamide's amino group (observed in both **1B** and **5B**). Furthermore, consistent with observations for the A series, a pyridine substituent, as in the case of **5B**, facilitates the bridging of the DEET‐ and the sIC‐ sites. In this case, the meta‐positioned nitrogen of the pyridine forms a direct H–bond interaction with Phe123 at the C‐terminal loop.

In silico docking results at the sIC‐binding site were also encouraging revealing that compounds use their pharmacophores in the optimal way to bridge interactions between the DEET‐ and the sIC‐sites. Notably, the critical pi–pi interaction with Trp114 (DEET‐binding site) is consistently preserved upon docking both series of analogs. Furthermore, compounds of the A series closely mimic the Icaridin binding motif at the sIC‐site, either contacting via H‐bonding the conserved water (i.e., **2A**) or establishing direct interaction with the C‐terminal loop and particularly Phe123 as shown for the analog **4A**. Additionally, **4A** stabilizes further its binding via a pi–pi interaction between its pyridine ring and the imidazole side chain of His111 (Figure 2B).

Analogs of the B series bound at sIC‐site, retain the same binding motif as compared with their binding poses at the DEET‐site. Thus, they consistently orient the sulfonamide functionality toward the DEET‐site enabling H‐bonding with key residues such as Ala88 (i.e., compound **1B**) or the side chain of His77 (i.e., compounds **2B** and **5B**). This latter interaction was also observed in the crystal structure of AgamOBP1:Icaridin, representing the second distinct binding mode of Icaridin at DEET‐site. Moreover, they successfully bridge interactions between the two sites through directly contacting Phe123 at sIC‐site (i.e., **1B** via its NH group) or H‐bonding with the conserved water at the sIC‐site ‐mimicking the carbonyl moiety of Icaridin‐ via the methoxy substitution in **2B** or the meta pyridine nitrogen in **5B** (Figure 2B).

**Figure 3:**
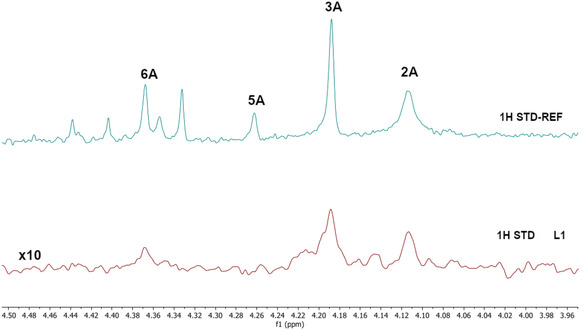
Bottom. ^1^H STD spectrum (*N*‐CH_2_‐Aryl resonances region) of the dynamic library L1 prepared under thermodynamic control from *p*‐toluidine (A) and aldehydes (**2**, **3**, **5**, **6**), in the presence of AgamOBP1 and subsequent reduction by NaBH_3_CN. Amines **2A**, **3A**, and **6A** are identified as binders, while **5A** shows no binding. The ^1^H STD‐REF NMR spectrum of L1 is also depicted (top).

Thus, two small DCLs were prepared under thermodynamic control consisting of *p*‐toluidine (A series) or 4‐(2‐aminoethyl)benzene sulfonamide (B series) (6 μL, 200 mM stock solution in DMSO‐d_6_) with a mixture of aldehydes **1–7** (1.2 μL for each aldehyde, 200 mM stock solution in DMSO‐d_6_) added to phosphate buffer in D_2_O (577.2 μL, pH = 6.1). The DCLs were kept at ambient temperature for three days producing the two libraries of imines DCL1 (**1A′–7A′**) and DCL2 (**1B′–7B′**), respectively. Subsequently, addition of AgamOBP1 to the equilibrated DCL1 and DCL2 amplified the formation of binders. After 24 h, reduction of the DCLs using NaBH_3_CN halted the reaction affording the final amines (**1A–7A**, **1B–7B**) (Table 1). The use of NaBH_3_CN enabled the smooth reduction of the imine double bond without affecting the carbonyl groups of the protein. ^1^H STD NMR spectroscopy was performed on the reduced DCLs. Unfortunately, the overlap of the *N‐*CH_2_‐Aryl resonances prohibited the unambiguous identification of the final amines binders of the two DCLs. Thus, we opted to prepare four sub‐libraries based on the *N‐*CH_2_‐Aryl resonances as follows: a) Library 1 (L1) included *p*‐toluidine (amine A) and aldehydes **2**, **3**, **5** and **6**; b) Library 2 (L2) comprised amine A and aldehydes **1**, **4** and **7**; c) Library 3 (L3) included 4‐(2‐aminoethyl) benzenesulfonamide (amine B) and aldehydes **1**, **3**, **5**, and **7**; and d) Library 4 (L4) comprised amine B and aldehydes **2**, **4**, and **6**. Gratifyingly, ^1^H STD NMR spectroscopy applied in the pre‐equilibrated L1 in the presence of AgamOBP1 identified the binding of the final amines **2A**, **3A**, and **6A** but not of **5A** (**Figure** **3**).

Unfortunately, analysis of L2, L3, and L4 by ^1^H STD NMR spectroscopy was inconclusive due to solubility issues of the library components in the buffer solution especially for L3 and L4 containing amine B.

### 2.2 Synthesis of Amines 1A‐7A and 1B‐7B and ^1^H STD NMR Spectroscopy Studies

**Scheme 1:**
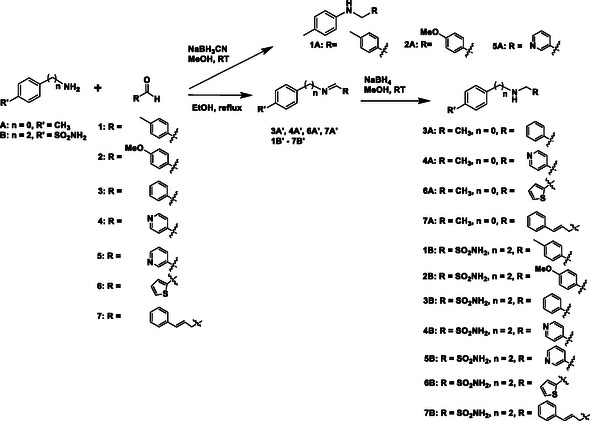
Synthesis of amines **1A–7A** and **1B–7B**.

Since our approach to employ pDCC in conjunction with ^1^H STD NMR spectroscopy was proven to be problematic for libraries L2–L4, we changed our strategy to a more traditional medicinal chemistry approach and proceeded to the synthesis of all secondary amines **1A–7A** and **1B–7B** as shown in **Scheme** **1**. Amines **1A**, **2A**, and **5A** were prepared from *p*‐toluidine and 4‐methylbenzaldehyde or 4‐methoxybenzaldehyde or 3‐pyridinecarboxaldehyde in the presence of NaBH_3_CN in methanol, respectively. However, this one pot reaction did not give good yields for the remaining desired amines, and we opted for a two‐step sequence. Thus, imines **3A′**, **4A′**, **6A′**, **7A′**, and **1B′–7B′** were prepared from p‐toluidine (**A**) or 4‐(2‐aminoethyl)benzenesulfonamide (**B**) and the appropriate aldehyde in refluxing ethanol. The pure imines were obtained via precipitation from ether and were treated with NABH_4_ in methanol to afford the desired amines **3A**, **4A**, **6A**, **7A**, and **1B–7B** after purification by flash column chromatography (Scheme 1).

**Figure 4:**
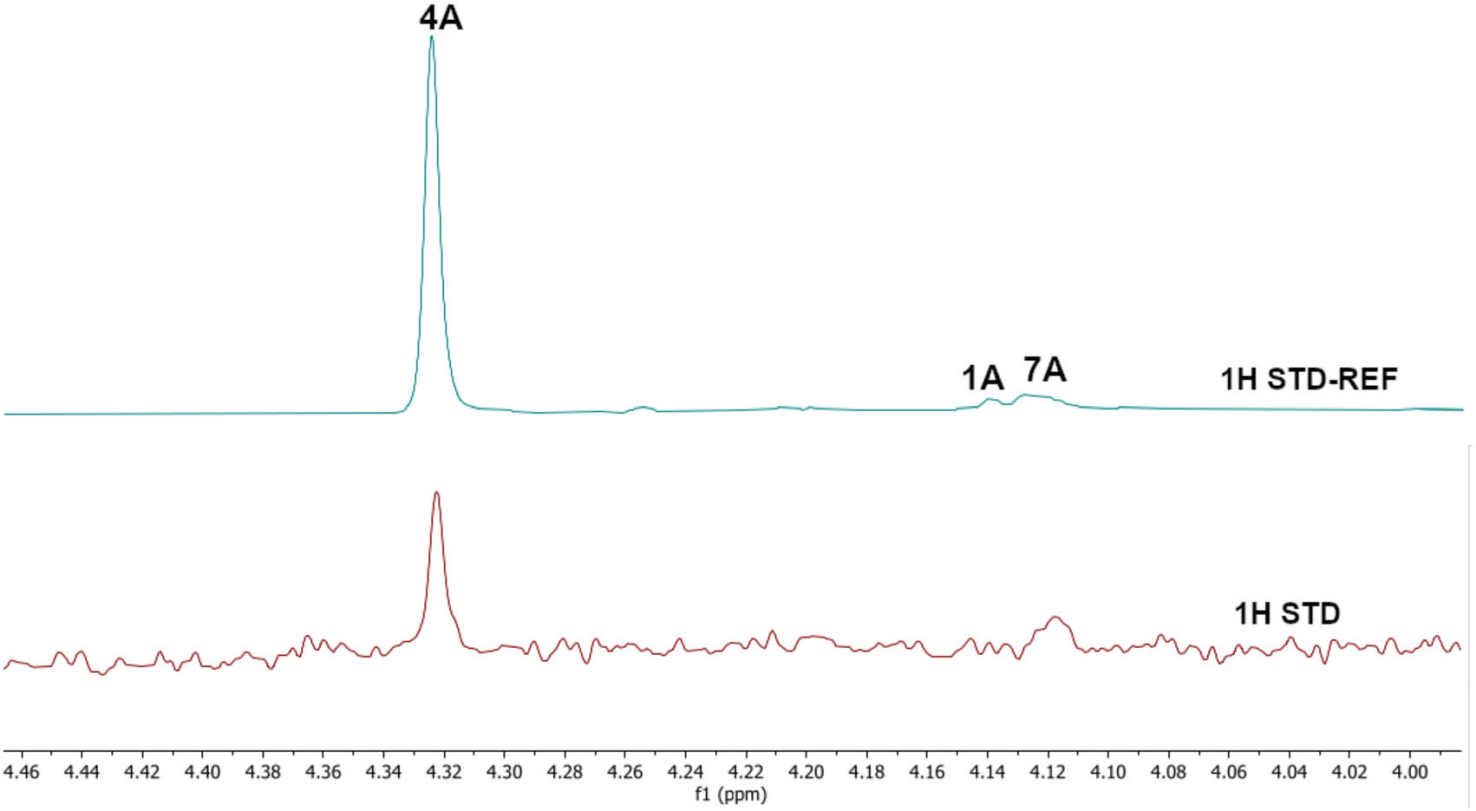
Interaction of AgamOBP1 with an equimolar mixture of the amines **1A**, **4A** and **7A.** The bottom panel shows the ^1^H STD NMR spectrum (*N*‐CH_2_‐Aryl resonances region) of the mixture in the presence of AgamOBP1 identifying **4A** as a strong binder. The ^1^H STD reference spectrum of the mixture is also depicted (top).

^1^H STD NMR spectroscopy was used in the presence of AgamOBP1 to select the amines that show binding interactions with the protein. The binding of amines **2A**, **3A**, **6A**, **5A** against AgamOBP1 was already studied in the context of dynamic library L1 above. Thus, based on the non‐overlapping *N*‐CH_2_‐Aryl proton resonances we prepared equimolar mixtures of amines **1A**, **4A**, and **7A**; of amines **1B**, **3B**, **5B**, and **7B**; and of amines **2B**, **4B** and **6B** which were further investigated for their interaction with AgamOBP1 by ^1^H STD NMR (**Figure** **4**, **5**–**6**). Amines **2A**, **3A**, **5A**, and **6A** were not studied further with ^1^H STD NMR spectroscopy in the presence of AgamOBP1 since L1 gave us promising results (Figure 3).

**Figure 5:**
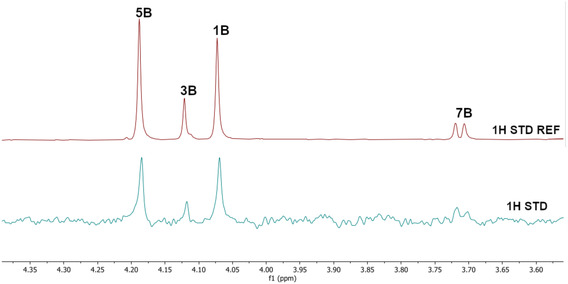
Interaction of AgamOBP1 with an equimolar mixture of amines **1B**, **3B**, **5B**, **7B**. The bottom panel shows the ^1^H STD NMR (*N*‐CH_2_‐Aryl resonances region) of the mixture in the presence of AgamOBP1 identifying the binding of the **1B**, **5B** and **3B** (weak interaction) while **7B** shows negligible interaction. The ^1^H STD reference spectrum of the mixture is also depicted (top).

**Figure 6:**
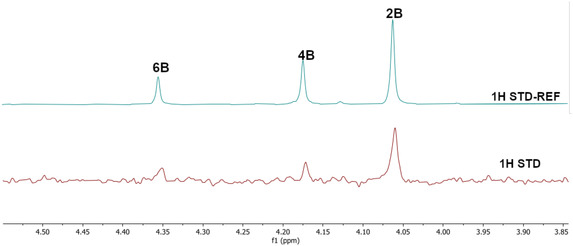
Interaction of AgamOBP1 with the equimolar mixture of the amines **2B**, **4B** and **6B**. The bottom panel shows the ^1^H STD NMR (*N*‐CH_2_‐Aryl resonances region) of the mixture in the presence of AgamOBP1 identifying the strong binding of the **2B** while **4B** and **6B** show minimal interactions. The ^1^H STD reference spectrum of the mixture is also depicted (top).

As shown in Figure 4 among **1A**, **4A**, and **7A**, only the pyridine‐substituted **4A** interacted strongly with the protein. A weak interaction was also observed for **7A**, although solubility issues may have affected both **1A** and **7A,** as indicated by their low signal intensity in the ^1^H STD‐REF spectrum.

^1^H STD NMR spectroscopy applied using the equimolar mixture of **1B**, **3B**, **5B**, and **7B** in the presence of AgamOBP1 indicated a strong interaction with the protein of **1B** substituted by a *p‐*methylphenyl group and **5B** bearing a meta‐substituted pyridine ring, a weaker interaction of **3B** possessing a phenyl group, while **7B** bearing a styryl group exhibited negligible interaction (Figure 5).

Finally, ^1^H STD NMR applied using an equimolar mixture of **2B**, **4B**, and **6B** in the presence of the protein revealed that **2B** possessing a *p*‐methoxyphenyl group displays a strong interaction with AgamOBP1, while the interaction of **4B** bearing a *para‐*substituted pyridine is weaker, while the interaction of **6B** substituted by thiophene is practically negligible (Figure 6).

Thus, the ^1^H STD experiments described above indicated amines **2A**, **3A**, **4A**, **6A**, **1B**, **2B**, and **5B** as binders of AgamOBP1.

### 2.3 Evaluation of the Binding of Amines **2A**, **3A**, **4A**, **6A**, **1B**, **2B**, and **5B** to AgamOBP1

In order to confirm the binding to AgamOBP1 of the hits showcased by the ^1^H STD NMR studies, a fluorescent probe displacement assay was employed. Thus, amines **2A**, **3A**, **4A**, **6A**, **1B**, **2B**, and **5B** were evaluated for their ability to displace the fluorescence probe *N*‐phenyl‐1‐naphthylamine (1‐NPN) from the AgamOBP1 binding site. In particular, amines **2A**, **3A**, **4A**, and **6A** were the most potent exhibiting higher competition (>25% displacement) compared with the reference repellents Icaridin and DEET (20.14% and 20.54% displacement, respectively). Conversely, amines **1B**, **2B**, and **5B** were not as active (% displacement ranging between 17.87 and 24.77) (**Table** **2** and Figure S1, Supporting Information).

**Table 2:** % displacement of 1‐NPN; from the AgamOBP1 binding site by amines **2A, 3A, 4A, 6A, 1B, 2B**, and **5B**, selected via ^1^H STD NMR spectroscopy and calculated vapor pressure values. For comparison, values for the reference repellents icaridin and DEET are included. Percentage displacement values are expressed as mean ± standard deviation (SD) from independent experiments performed in triplicate.

Compound	% 1‐NPN displacement ± SD	Calculated vapor pressure 25 °C [mmHg]
Icaridin	20.14 ± 0.70	8.26·10^−6^
DEET	20.54 ± 0.45	3.31·10^−3^
**2A**	29.56 ± 0.41	4.22·10^−5^
**3A**	26.44 ± 0.69	7.08·10^−4^
**4A**	25.91 ± 0.41	1.57·10^−4^
**6A**	34.21 ± 0.24	1.34·10^−4^
**1B**	17.87 ± 0.43	1.87·10^−8^
**2B**	24.77 ± 0.55	8.05·10^−9^
5B	20.56 ± 0.64	2.85 10^−8^

### 2.4 Prediction of Volatility

The lack of sufficient volatility could result in the absence of repellency. Therefore, we proceeded to the calculation of the vapor pressure (VP)^[^ ^51^ ^]^ of the hits with confirmed binding affinity for AgamOBP1 (Table 2). The VP values of **2A**, **3A**, **4A**, and **6A** ranged between those of Icaridin (8.26·10^−6^ mm Hg) and DEET (3.31 × 10^−3^), thus meeting the volatility criterion for their use as repellents. In contrast, the VP values of **1B**, **2B**, and **5B** at 1.87 × 10^−8^ mm Hg, 8.05 × 10^−9^ mm Hg, and 2.85 × 10^−8^ mm Hg, respectively, are below the threshold for significant volatility (7.5 × 10^−7^ mm Hg) as defined by Spicer et al.^[^ ^44^ ^,^ ^52^ ^]^ As a result, amines **1B**, **2B**, and **5B** were not selected for the subsequent repellency behavioral tests.

### 2.5 Assessment of Repellent Activity

Amines **2A**, **3A**, **4A**, and **6A** possessing the highest % displacement values and satisfactory volatility prediction values were further evaluated for their repellent efficacy using an arm‐in‐cage repellency test against *Aedes albopictus* in two doses 0.2 μL cm^−^^2^ (low) and 0.4 μL cm^−^^2^ (high). All the tested compounds showed significant repellent activity and can be considered promising scaffolds for further optimization studies (**Table** **3** and Figure S2, Supporting Information). Specifically, compound **4A** acts as a middle repellent at the low dose and as a DEET‐like repellent at the high dose. Compound **6A** is a middle repellent at low dose and a strong repellent at the high dose, followed in potency by **2A** which is a middle to strong repellent in the high dose. The weakest repellent among the series is **3A** which displays a 53% protection at the high‐dose. Interestingly, the pyridine derivative **4A** is the most potent of the compounds tested despite the fact that **6A** possessing a thiophene moiety shows higher % 1‐NPN displacement efficacy and lower calculated VP. Consistent with the repellent potency results amine **4A** exhibited the highest *in silico* binding score among the tested compounds for both DEET‐ and sIC‐sites.

**Table 3:** % Repellence of the tested compounds in 5 min arm‐in‐cage experiments against *Ae. albpopictus.*

Compound	Structure	Mean repellency index [%] ± SEa)	
		0.2 μL cm^−2^	0.4 μL cm^−2^
**2A**	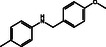	32.6 ± 11.6 (d)	72.25 ± 5.6 (c)
**3A**	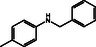	15.3 ± 5.0 (c)	53 ± 13.6 (d)
**4A**	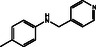	60.1 ± 4.7 (b)	92.3 ± 4.3 (b)
**6A**	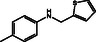	68.3 ± 7.7 (b)	77.25 ± 4.6 (c)
DEETb)		95.8 ± 2.6 (a)	99.8 ± 0.5 (a)

a) For each dose, different letters (a, b, c, d) indicate statistically significant differences between tested compounds (*p* < 0.05), Mann–Whitney *U* test with Bonferroni correction (adjusted *p* values *a* = 0.005).

b) DEET, *N,N*‐diethyl‐3‐methylbenzamide.

## 3 Conclusion

Toward the discovery of next generation insect repellents, protein‐directed dynamic combinatorial chemistry (pdDCC) coupled to saturation‐transfer difference (STD) spectroscopy was initially employed using the malaria vector *Anopheles gambiae* olfactory protein AgamOBP1. Imine formation and subsequent reduction with NaBH_3_CN to the corresponding amines was employed as the chemistry for the dynamic libraries. The design of the library members was based on in silico studies utilizing the previously identified binding sites of DEET and Icaridin (the DEET‐site and the sIC(Icaridin)‐site, respectively). Only one dynamic library was studied using this approach since solubility issues of the other library components resulted in low signal to noise ratios in the ^1^H STD NMR spectra. Thus, the 14 designed amines, putative products from the dynamic libraries, were synthesized and equimolar mixtures were studied using ^1^H STD spectroscopy in the presence of AgamOBP1. The identified binders, amines **2A**, **3A**, **4A**, **6A**, **1B**, **2B**, and **5B** were further confirmed by fluorescence competition assays while prediction of volatility further prioritized amines **2A**, **3A**, **4A**, and **6A** for behavioral studies. Amines **2A**, **3A**, **4A**, and **6A** showed significant repellent activity albeit, lower than that of DEET. The most potent derivative was **4A** (4‐methyl‐N‐(pyridin‐4‐ylmethyl)aniline) acting as a DEET‐like repellent at the high dose and a middle repellent at the low dose. In conclusion our strategy, applied for the first time using AgamOBP1 as a protein template, resulted in promising hits suitable for further optimization studies towards targeted and efficient repellents with potential application against vector‐borne diseases.

## 4 Experimental Section

4.1

4.1.1

##### In Silico Methods

All amines that composed the examined compound library were primarily sketched and then were prepared at the optimum pH = 6.5 ± 0.5, using LigPrep^[^ ^53^ ^]^ program of MAESTRO.^[^ ^54^ ^]^ Also, the physicochemical properties (Table S3, Supporting Information) of the tested amines were predicted by QikProp^[^ ^55^ ^]^ program of MAESTRO. Subsequently, molecular docking simulations were performed to all compounds applying the Extra Precision (XP) mode of Glide^[^ ^56^ ^,^ ^57^ ^]^ to identify the favorable binding configurations of the compounds. During the docking process, the AgamOBP1 protein was essentially frozen and the tested amines adapted conformational flexibility. For this scope, the crystal structures of AgamOBP1 complexed with DEET (PDB ID:3N7H)^[^ ^40^ ^]^ and Icaridin (PDB ID:5EL2)^[^ ^37^ ^]^ were subjected to Protein Preparation and a grid box with dimensions 10 × 10 × 10 Å was generated, respectively.^[^ ^58^ ^,^ ^59^ ^]^ In the case of AgamOPB1 co‐crystallized with Icaridin (PDB ID:5EL2), the possible orientations and interactions of the compound library were evaluated into the sIC‐binding site. The docking poses were visually inspected and their binding modes were analyzed.

##### ^1^H STD NMR Spectroscopy

All ^1^H STD NMR experiments were performed at 25 °C on a Varian 600 MHz spectrometer using a 1H{15N‐31P} 5 mm PFG Automatable Triple Resonance probe. Experiments were run using the pulse sequences provided by the Varian BioPack library. STD NMR experiments were run using the dpfgse_satxfer sequence and STD‐REF experiments with the dpfgse_satxfer2 sequence. The ^1^H STD NMR experiments were recorded with a spectral width of 12,019 Hz, 8192 complex data points, and 20,000 scans. Selective on‐resonance irradiation frequency was set to 0.35 ppm and the off‐resonance irradiation frequency was applied at 30 ppm. The saturation scheme consisted of a train of 50 ms Gauss‐shaped pulses separated by a 0.1 ms delay with total duration of 2.5 s.

The bound amines were identified by analyzing the methylene protons of the *N*‐CH_2_‐Aryl group in the ^1^H STD NMR spectra. The identification was assisted by control ^1^H NMR experiments of the final amines.

Amines or aldehydes stock solutions were prepared in deuterated dimethylsulfoxide (DMSO‐d_6_) at a concentration of 200 mM. AgamOBP1 stock solution was prepared in phosphate buffer at a concentration of 0.2 mM.

The mixtures of amines **1A–7A** and **1B–7B** with AgamOBP1 were prepared by mixing a protein stock solution with a concentration of 0.2 mM with stock solutions of each ligand with a concentration of 200 mM resulting protein: ligand ratio of 1:100.

Phosphate buffer solution was prepared by adding to a solution of 33.44 mg K_2_HPO_4_ in 1.92 mL D_2_O a solution of 109.96 mg KH_2_PO_4_ in 8.08 mL D_2_O up to 6.5 pD. Since deuterated water is used, the pH value is corrected based on the equation pD = pH + 0.4.

##### DCL Preparation

*p*‐Toluidine (amine A) (6 μL, 200 mM stock solution in DMSO‐d_6_) and aldehydes **2**, **3**, **5**, and **6** (1.2 μL for each aldehyde, 200 mM stock solution in DMSO) or aldehydes **1**, **4**, and **7** (1.2 μL for each aldehyde, 200 mM stock solution in DMSO) were added to phosphate buffer in D_2_O (577.2 μL, pH = 6.1).

The DCLs were kept at ambient temperature for three days and AgamOBP1 (12 μL, 0.2 mM stock solution in phosphate buffer in D_2_O, pH = 6.3) was then added to the equilibrated DCL, and the mixture stands for one more day at ambient temperature, and then NaBH_3_CN (0.02 mmol) was added, and the mixture was left standing for day prior to ^1^H STD NMR spectroscopy analysis.

4‐(2‐Aminoethyl)benzenesulfonamide (amine B) (6 μL, 200 mM stock solution in DMSO‐d_6_) and aldehydes **1**, **3**, **5**, and **7** (1.2 μL for each aldehyde, 200 mM stock solution in DMSO) or aldehydes **2**, **4**, and **6** (1.2 μL for each aldehyde, 200 mM stock solution in DMSO) were added to phosphate buffer in D_2_O (577.2 μL, pH = 6.1).

The DCLs were kept at ambient temperature for 3 days and AgamOBP1 (12 μL, 0.2 mM stock solution in phosphate buffer in D_2_O, pH = 6.3) was then added to the equilibrated DCL, and the mixture stands for one more day at ambient temperature, and then NaBH_3_CN (0.02 mmol) was added, and the mixture was left standing for day prior to ^1^H STD NMR spectroscopy analysis.

The final concentrations of the amine A or amine B and each aldehyde in the DCL were 2 mM and 0.4 mM, respectively, in order to yield 0.4 mM of each possible product. The final concentration of AgamOBP1 was 0.004 mM, resulting a protein:ligand ratio of 1:100.

##### Preparation of the Mixtures of Amines **1A**, **4A**, **7A**, and **1B–7B** for ^1^H STD NMR Spectroscopy

The screening of the final amines against AgamOBP1 was performed using a mixture of **1A**, **4A**, and **7A**; of amines **1B**, **3B**, **5B**, and **7B**; and of amines **2B**, **4B**, and **6B** (1.2 μL, 200 mM stock solution in DMSO for each final amine) that were added to phosphate buffer in D_2_O until the final volume of 600 μL (584 μL, pH = 6,1). AgamOBP1 (12 μL, 0.2 mM stock solution in phosphate buffer in D_2_O, pH = 6.3) was then added to each mixture of amines.

The final concentration of each amine was 0.4 mM, and the final concentration of AgamOBP1 was 0.004 mM, resulting in a protein:ligand ratio of 1:100.

##### Bacterial Expression and Purification of Recombinant AgamOBP1 Protein

The pET22b(+)‐AgamOBP1 expression plasmid was transformed into *E. coli* Origami B (DE3) competent cells (Novagen) to express the full‐length protein lacking its N‐terminal signal peptide (residues 20–144 of UniProt entry Q8I8T0) as previously described.^[^ ^40^ ^]^ The clarified cell lysate was initially applied to a HiTrap Q FF column (GE Healthcare), and elution was carried out using a 0–500 mM NaCl gradient. Fractions containing the target protein were further purified on a Resource Q column (GE Healthcare) with a linear gradient of 0–300 mM NaCl. This purification step was followed by size‐exclusion chromatography on a Superdex 75 column (GE Healthcare). The resulting highly purified protein fractions were desalted by dialysis and concentrated to 15 mg mLm^−1^ in 10 mM Tris‐HCl (pH 8.0).

##### One‐Point in Vitro Competitive Fluorescence Assay

The binding affinity of AgamOBP1 for the selected amines was evaluated indirectly by determining the displacement of the fluorescent probe N‐phenyl‐1‐naphthylamine (1‐NPN) from the protein by the ligand. Simultaneously, Icaridin and DEET were tested and used as controls. The probe was allowed to bind to the protein, and the maximal fluorescence intensity was measured in the absence and presence of the ligands. The emission spectra were obtained at 30 °C in a VarioSkan Flash plate reader (Thermo Scientific, USA) using black 96‐well plates (Greiner, Bio‐One). The probe was excited at 337 nm, and emission spectra were recorded between 390 and 500 nm. In the presence of AgamOBP1, the maximum fluorescence emissions were observed at 408 nm. Ligand binding was performed in a final volume of 200 µl, in 50 mM Tris‐HCl, pH 8.0. AgamOBP1 was assayed at a final concentration of 2 μM in the presence of 10 μM 1‐NPN and in the absence and the presence of 5 μM ligand. Fresh stock solutions of 1‐NPN, Icaridin, DEET, and the selected amines were prepared in 100% DMSO. A final concentration of 2% DMSO was maintained in all dilutions. Three independent experiments were run for each 1‐NPN/ligand combination.

##### Rearing of *Ae. albopictus* in the Laboratory

Adult *Ae. albopictus* mosquitoes were obtained from a laboratory colony which was maintained at 25 ± 2 °C, 80% relative humidity, and photoperiod of 16:8 h light/dark (L/D), in the laboratory of the Benaki Phytopathological Institute, Kifissia, Greece.^[^ ^60^ ^]^ For oviposition, plastic beakers containing 100 ml of water and strips of moistened filter paper were placed in the cage. The eggs were kept damp for a few days before being transferred to pans for hatching. The larvae were reared in cylindrical enamel pans filled with tap water, with ≈400 larvae/pan. They were fed in excess with powdered fish food (JBL Novo Tom 10% Artemia) until the emergence of adults. Adult mosquitoes were collected using a mouth aspirator and transferred to the rearing cage. Females were fed with fresh chicken blood using Hemotek blood‐feeding system.

##### Repellent Activity Bioassay

The repellent activity of the selected compounds was assessment using human landing counts as described in previous studies.^[^ ^60^ ^,^ ^61^ ^]^ The bioassay was conducted in a cage (33 × 33 × 33 cm) with a 32 × 32 mesh containing 100 adult mosquitoes (1:1 sex ratio,), aged 5‐ to 10‐days and starved for 12 h at 25 ± 2 °C, and 70–80% relative humidity. A plastic glove with a 5 × 5 cm opening was used for all the bioassays. Testing materials were applied on Whatman chromatography paper (24 cm^2^) at two doses: 0.2 and 0.4 μL cm^−^^2^. DEET was used as positive control, while each test compound was dissolved in dichloromethane (DCM). Control treatments (with only DCM) were also included. The proposed protocol suggests an optimum dose of ≈1 mg cm^−^^2^ for each compound, which is commonly used in efficacy tests for repellents.^[^ ^62^ ^]^ The total exposure time was 5 min. Each treatment was repeated eight times and four human volunteers were used.

##### Determination of Repellency Indices (RI%)

Landing numbers were converted to repellency indices (RI ± SE) using the following equation:

(1) RI%=[1−(T/C)]×100=[(C−T)/C]×100

where C is the number of landings and/or probings in the control arm (C) and T is the number of landings in the treatment of the same individual at the end of the experiment (5 min).

##### Statistical Analysis

Data concerning the repellency indices were analyzed using the Kruskal–Wallis test.^[^ ^63^ ^]^ When significant differences were detected, Mann–Whitney *U* tests were carried out for pair‐wise comparison with a Bonferroni correction for adjustment of *p* values.

##### Calculation of Vapor Pressure

The vapor pressures of the tested compounds were estimated using the Boiling Point/Vapor Pressure module as implemented in the ACD/I‐Lab 2.0 suite (ACD) https://ilab.acdlabs.com/iLab2/.

##### Chemistry: General

The chemicals required were obtained from Sigma–Aldrich, Alfa Aesar, Acros Organics, and were used as such. All reactions were conducted under a nitrogen atmosphere. The flash column chromatography was carried out on Merck silica gel (200–400 mesh). ^1^H NMR spectra of the compounds **4A**, **6A**, **7A**, **4A′**, **6A′**, **7A′**, **1B–7B**, **1B′**, **3B′**, **4B′**, **5B′**, **6B′**, **7B′** were obtained with Varian 300 MHz spectrometer using DMSO‐d_6_ as solvent. ^1^H NMR spectra of the compounds **2A**, **3A**, **2B′** were obtained with Varian 300 MHz spectrometer using CDCl_3_ as solvent. ^1^H and ^13^C NMR spectra of the compounds **1A**, **5A** were obtained with Varian 600 MHzspectrometer using CDCl_3_ as solvent. Chemical shifts in ^1^H NMR spectra are reported in parts per million (ppm, *δ*) relative to the central line of the solvent signal (*δ* = 2.50 ppm for DMSO‐d_6_ and *δ* = 7. 26 ppm for CDCl_3_). Mass spectra were obtained on a LC‐MS Fleet, Thermo spectrometer where the ionization of the substances was done by electrospray technique (ESI, Electron Spray Ionization). MeOH (LC‐MS grade) was used as a solvent. High‐resolution mass spectra (HR‐MS) were obtained on a UHPLC LC‐MS Orbitrap Velos‐Thermo mass spectrometer. Flash column chromatography (FCC) was performed on silica gel 60 (230–400 mesh, Merck KGaA, Darmstadt, Germany) and thin‐layer chromatography (TLC) on pre‐coated silica gel glass plates 60 F254 (0.2 mm, Merck) or neutral aluminum oxide precoated plates F254 (0.2 mm, Merck). Spots were visualized with UV light at 254 nm and phosphomolybdic acid stain (PMA, 10% in absolute ethanol).^[^ ^64^ ^,^ ^65^ ^]^

##### Chemistry: General Procedure A (GPA): Synthesis of the Amines **1A**, **2A**, **5A**.

To an ice cold solution of *p*‐toluidine (10 eq) in methanol (0.2 m) aldehyde **1** or **2** or **5** was added (1 eq) and NaBH_3_CN (3 eq). The reaction mixture was then stirred at ambient temperature, for 24 h. Upon completion of the reaction, the solvent was evaporated under pressure to dryness. The product was obtained after FCC purification.

##### Chemistry: General Procedure B (GPB)—Synthesis of the Imines **2A′**, **3A′**, **4A′**, **6A′**, **7A′**, **1B′–7B′**

To a solution of each amine (**A** or **B**) (1 eq) in absolute ethanol (0.04 m), aldehyde was added (1.1 eq). The reaction mixture was then refluxed for 24 h. Upon completion of the reaction, the solvent was evaporated under vacuum to dryness. Pure imine was precipitated from ether.

##### Chemistry: General Procedure C (GPC)—Synthesis of the Amines **2A**, **3A**, **4A**, **6A**, **7A**, **1B–7B**

To a solution of each imine (1 eq) in methanol (0.4 m) NaBH_4_ (3 eq) was added in 30 min. The reaction mixture was then stirred at ambient temperature, for 3 to 24 h. Upon completion of the reaction, the solvent was evaporated under vacuum to dryness, the solid was dissolved in DCM, and the solution was washed with brine, dried over anhydrous Na_2_SO_4_, and evaporated to dryness to afford the pure products.

##### Chemistry: 4‐Methyl‐N‐(4‐Methylbenzyl)aniline (**1A**)

Compound **1A** was prepared according to GPA by reaction of *p*‐toluidine (1.07 g, 10 mmol) with 4‐methylbenzaldehyde (121 mg, 1 mmol) in methanol (5 mL) in the presence of NaBH_3_CN (188 mg, 3 mmol). Flash column chromatography (silica gel, PE 40–60 °C/diethyl ether 94:6) afforded compound **1A** (154 mg, 73%) as a dark yellow solid. Rf = 0.45 (silica gel, PE 40–60 °C/diethyl ether 85:15); MS (ESI), m/z 212,00 [M + H]^+^; ^1^H NMR (600 MHz, CDCl_3_): *δ* 7.25 (d, *J* = 7.8 Hz, 2H, ArH), 7.14 (d, *J* = 7.8 Hz, 2H, ArH), 6.98 (d, *J* = 8.2 Hz, 2H, ArH), 6.56 (d, *J* = 8.2 Hz, 2H, ArH), 4.26 (s, 2H, CH_2_), 2.34 (s, 3H, CH_3_), 2.23 (s, 3H, CH_3_).

##### Chemistry: *N*‐(4‐Methoxybenzyl)‐4‐Methylaniline (**2A**)

Compound **2A** was prepared according to GPA by reaction of *p*‐toluidine (1.07 mg, 10 mmol) with 4‐methoxybenzaldehyde (136 mg, 1 mmol)) in methanol (5 mL) in the presence of NaBH_3_CN (188 mg, 3 mmol). Flash column chromatography (silica gel, PE 40–60 °C/diethyl ether 9:1) afforded compound **2A** (119 mg, 52%) as yellow solid. Rf = 0.2 (silica gel, PE 40–60 °C/diethyl ether 85:15); MS (ESI), m/z 227,830 [M + H]+; ^1^H NMR (300 MHz, CDCl_3_) *δ* 7.29 (d, *J* = 8.4 Hz, 2H, ArH), 6.99 (d, *J* = 8.2 Hz, 2H, ArH), 6.88 (d, *J* = 8.5 Hz, 2H, ArH), 6.57 (d, *J* = 8.2 Hz, 2H, ArH), 4.23 (s, 2H, CH_2_), 3.80 (s, 3H, OCH_3_), 2.24 (s, 3H, CH_3_).

##### Chemistry: *N*‐Benzyl‐4‐Methylaniline (**3A**)

Compound **3A** was prepared according to GP A by reaction of p‐toluidine (1.07 mg, 10 mmol) with benzaldehyde (106 mg, 1 mmol)) in methanol (5 mL) in the presence of NaBH_3_CN (188 mg, 3 mmol). Flash column chromatography (silica gel, PE 40–60 °C/diethyl ether 97:3) afforded compound **3A** (175 mg, 88%) as light brown oil. Rf = 0.47(Aluminum oxide, hexane/ethyl acetate 95:5); MS (ESI), m/z 198,050 [M + H]^+^; ^1^H NMR (300 MHz, CDCl_3_) *δ* 7.35–7.14 (m, 5H, ArH), 6.91 (d, *J* = 8.2 Hz, 2H, CH_2_), 6.49 (d, *J* = 8.4 Hz, 2H, CH_2_), 4.24 (s, 2H, CH_2_), 2.16 (s, 3H, CH_3_).

##### Chemistry: 4‐Methyl‐N‐(pyridin‐3‐Ylmethyl)aniline (**5A**)

Compound **5A** was prepared according to GPA by reaction of *p*‐toluidine (1.07 g, 10 mmol) with 3‐pyridinecarboxaldehyde (107 mg, 1 mmol) in methanol (5 mL) in the presence of NaBH_3_CN (188 mg, 3 mmol). Flash column chromatography (silica gel, PE 40–60 °C/diethyl ether 3:7) afforded compound **5A** (138 mg, 70%) as a dark yellow oil. Rf = 0.29 (silica gel, PE 40–60 °C/diethyl ether 2:8); MS (ESI), m/z 199,170 [M + H]^+^; ^1^H NMR (600 MHz, CDCl_3_): *δ* 8.63 (s, 1H, ArH), 8.52 (d, *J* = 3.0 Hz, 1H, ArH), 7.71 (d, *J* = 7.2 Hz, 1H, ArH), 7.28–7.26 (m, 1H, ArH), 6.99 (d, *J* = 7.7 Hz, 2H, ArH), 6.55 (d, *J* = 7.7 Hz, 2H, ArH), 4.34 (s, 2H, CH2), 2.24 (s, 3H, CH_3_).

##### Chemistry: 1‐(pyridin‐4‐Yl)‐N‐(p‐Tolyl)methanimine (**4A′**)

Compound **4A′** was prepared according to GPB by reaction of *p*‐toluidine (107 mg, 1 mmol) with 4‐pyridinecarboxaldehyde (118 mg, 1,1 mmol). Pure imine **4A′** was obtained (192 mg 98%) as yellow crystalline solid. Rf = 0.46 (Aluminum oxide, hexane/ethyl acetate 1:1); ^1^H NMR (300 MHz, DMSO‐d_6_) *δ* 8.74 (d, J = 5.0 Hz, 2H, ArH), 8.70 (s, 1H, N = CH), 7.84 (d, *J* = 5.0 Hz, 2H, ArH), 7.26 (bs, 4H, ArH), 2.34 (s, 3H, CH_3_).

##### Chemistry: 1‐(thiophen‐2‐Yl)‐N‐(p‐Tolyl)methanimine (**6A′**)

Compound **6A′** was prepared according to GPB by reaction of *p*‐toluidine (107 mg, 1 mmol) with thiophene‐2‐carbaldehyde (123 mg, 1.1 mmol). Pure imine **6A′** was obtained (193 mg 96%) as dark brown solid. ^1^H NMR (300 MHz, DMSO‐d_6_) *δ* 8.77 (s, 1H, N = CH), 7.79 (d, *J* = 4.9 Hz, 1H), 7.66 (d, *J* = 2.4 Hz, 1H), 7.23–7.15 (m, 5H, ArH), 2.31 (s, 3H).

##### Chemistry: 3‐Phenyl‐*N*‐(*p*‐Tolyl)prop‐2‐en‐1‐Imine (**7A′**)

Compound **7A′** was prepared according to GPB by reaction of *p*‐toluidine (107 mg, 1 mmol) with cinnamaldehyde (145 mg, 1.1 mmol). Pure imine **7A′** was afforded (217 mg, 98%) as yellow crystalline solid. Rf = 0.46 (Aluminum oxide, hexane/ethyl acetate 1:1); ^1^H NMR (300 MHz, DMSO‐d_6_) *δ* 8.74 (d, *J* = 5.0 Hz, 2H, ArH), 8.70 (s, ^1^H, N = CH), 7.84 (d, *J* = 5.0 Hz, 2H, ArH), 7.26 (bs, 4H, ArH), 2.34 (s, 3H, CH_3_).

##### Chemistry: 4‐(2‐((4‐Methylbenzylidene)amino)ethyl)benzenesulfonamide **1B′**

Compound **1B′** was prepared according to GPB by reaction of 4‐(2‐aminoethyl)benzenesulfonamide (200 mg, 1 mmol) with 4‐methylbenzaldehyde (132 mg, 1.1 mmol). Pure imine **1B′** was obtained (196.4 mg, 65%) as white solid. MS (ESI), m/z 303.116 [M + H]^+^ **;** HRMS (ESI) calcd for C_16_H_19_O_2_N_2_S [M + H]^+^ 303.1162, found 303.1160; ^1^H NMR (300 MHz, DMSO‐d_6_) *δ* 8.24 (s, 1H, N = CH), 7.72 (d, *J* = 8.2 Hz, 2H, ArH), 7.59 (d, *J* = 8.0 Hz, 2H, ArH), 7.44 (d, *J* = 8.2 Hz, 2H, ArH), 7.25 (s, 2H, NH_2_), 7.24 (d, *J* = 8.0 Hz, 2H, ArH), 3.81 (t, *J* = 6.8 Hz, 2H, CH_2_), 3.00 (t, *J* = 7.1 Hz, 2H, CH_2_), 2.33 (s, 3H, CH_3_).

##### Chemistry: 4‐(2‐((4‐Methoxybenzylidene)amino)ethyl)benzenesulfonamide **2B′**

Compound **2B′** was prepared according to GPB by reaction of 4‐(2‐aminoethyl)benzenesulfonamide (200 mg, 1 mmol) with 4‐methoxybenzaldehyde (150 mg, 1.1 mmol). Pure imine **2B′** was obtained as off white solid (246 mg, 77%). MS (ESI), m/z 319.111 [M + H]^+^; HRMS (ESI) calcd for C_16_H_19_O_3_N_2_S [M + H]^+^ 319.1111, found 319.1110; ^1^H NMR (300 MHz, CDCl_3_) *δ* 8.02 (s, 1H, N = CH), 7.75 (d, *J* = 8.4 Hz, 2H, ArH), 7.55 (d, *J* = 8.7 Hz, 2H, ArH), 7.37–7.14 (m, 2H, ArH), 6.83 (d, *J* = 8.7 Hz, 2H, ArH), 5.94 (s, 2H, NH_2_), 3.76 (s, 3H, OCH_3_), 3.74 (t, *J* = 7.1 Hz, 2H, CH_2_), 2.97 (t, *J* = 7.1 Hz, 2H, CH_2_).

##### Chemistry: 4‐(2‐(benzylideneamino)ethyl)benzenesulfonamide **3B′**

Compound **3B′** was prepared according to GPB by reaction of 4‐(2‐aminoethyl)benzenesulfonamide (200 mg, 1 mmol) with benzaldehyde (117 mg, 1.1 mmol). Pure imine **3B′** was obtained as white solid (177 mg, 61%). ^1^H NMR (300 MHz, DMSO‐d_6_) *δ* 8.30 (s, 1H, N = CH), 7.74–7.69 (m, 4H, ArH), 7.46–7.43 (m, 5H, ArH), 7.26 (s, 2H, NH_2_), 3.84 (t, J = 7.1 Hz, 2H, CH_2_), 3.01 (t, *J* = 7.1 Hz, 2H, CH_2_).

##### Chemistry: (*E*)‐4‐(2‐((pyridin‐4‐Ylmethylene)amino)ethyl)benzenesulfonamide **4B′**

Compound **4B′** was prepared according to GPB by reaction of 4‐(2‐aminoethyl)benzenesulfonamide (200 mg, 1 mmol) with 4‐pyridinecarboxaldehyde (118 mg, 1.1 mmol). Pure imine **4B′** was obtained as off white solid (244 mg, 84%). MS (ESI), m/z 290.096 [M + H]^+^; HRMS (ESI) calcd for C_14_H_16_O_2_N_3_S [M + H]^+^ 290.0958, found 290.0958. ^1^H NMR (300 MHz, DMSO‐d_6_) *δ* 8.66 (d, *J* = 5.9 Hz, 2H, ArH), 8.34 (s, 1H, N = CH), 7.73 (d, *J* = 8.3 Hz, 2H, ArH), 7.64 (d, J = 5.9 Hz, 2H, ArH), 7.45 (d, J = 8.3 Hz, 2H, ArH), 7.27 (s, 2H, NH_2_), 3.90 (t, ***J*** = 6.9 Hz, 2H, CH2), 3.04 (t, *J* = 7.1 Hz, 2H, CH_2_).

##### Chemistry: 4‐(2‐((pyridin‐3‐Ylmethylene)amino)ethyl)benzenesulfonamide **5B′**

Compound **5B′** was prepared according to GPB by reaction of 4‐(2‐aminoethyl)benzenesulfonamide (200 mg, 1 mmol) with 3‐pyridinecarboxaldehyde (118 mg, 1.1 mmol). Pure imine **5B′** was obtained as white solid (268 mg, 93%). MS (ESI), m/z 290.096 [M + H]^+^ **;** HRMS (ESI) calcd for C_14_H_16_O_2_N_3_S [M + H]^+^ 290.0958, found 290.0961. ^1^H NMR (300 MHz, DMSO‐d_6_) *δ* 8.85 (d, *J* = 1.3 Hz, 1H, ArH), 8.63 (dd, *J* = 4.8, 1.5 Hz, 1H, ArH), 8.37 (s, 1H, N = CH), 8.09 (dt, *J* = 7.9, 1.7 Hz, 1H, ArH), 7.73 (d, *J* = 8.3 Hz, 2H, ArH), 7.49—7.44 (m, 3H, ArH), 7.27 (s, 2H, NH_2_), 3.87 (t, *J* = 7.0 Hz, 2H, CH_*2*_), 3.03 (t, *J* = 7.0 Hz, 2H, CH_2_).

##### Chemistry: (2‐((thiophen‐2‐Ylmethylene)amino)ethyl)benzenesulfonamide **6B′**

Compound **6B′** was prepared according to GPB by reaction of 4‐(2‐aminoethyl)benzenesulfonamide (200 mg, 1 mmol) with thiophene‐2‐carbaldehyde (123 mg, 1.1 mmol). Pure imine **6B′** was obtained as brown solid (254 mg, 86.5%). MS (ESI), m/z 295.056 [M + H]^+^; HRMS (ESI) calcd for C_14_H_16_O_2_N_3_S [M + H]^+^ 295.0569, found 295.0567; ^1^H NMR (300 MHz, DMSO‐d_6_) *δ* 8.40 (s, 1H, N = CH), 7.72 (d, J = 8.1 Hz, 2H, ArH), 7.65 (d, ***J*** = 4.9 Hz, 1H, ArH), 7.44–7.41 (m, 3H, ArH), 7.26 (bs, 2H, NH_2_), 7.14–7.11 (m, 1H, ArH), 3.77 (t, *J* = 7.0 Hz, 2H, CH2), 2.98 (t, *J* = 7.0 Hz, 2H, CH_2_).

##### Chemistry: 4‐(2‐(((1E, 2E)‐3‐Phenylallylidene)amino)ethyl)benzenesulfonamide **7B**′

Compound **7B′** was prepared according to GPB by reaction of 4‐(2‐aminoethyl)benzenesulfonamide (200 mg, 1 mmol) with cinnamaldehyde (145 mg, 1.1 mmol). Pure imine **7B′** was obtained as yellow solid (253 mg, 80.6%). MS (ESI), m/z 315.116 [M + H]^+^; HRMS (ESI) calcd for C_17_H_19_O_2_N_2_S [M + H]^+^ 315.1162, found 315.1161.^1^H NMR (300 MHz, DMSO‐d_6_) *δ* 8.03 (d, *J* = 8.6 Hz, 1H, N = CH), 7.73 (d, *J* = 8.2 Hz, 2H, ArH), 7.58 (d, ***J*** = 6.8 Hz, 2H, ArH), 7.49–7.25 (m, 7H, ArH, NH2), 7.06 (d, *J* = 16.1 Hz, 1H, CH), 6.89 (dd, J = 16.0, 8.6 Hz, 1H, CH), 3.74 (t, J = 6.9 Hz, 2H, CH2), 2.96 (t, J = 7.0 Hz, 2H, CH_s_).

##### Chemistry: 4‐Methyl‐N‐(pyridin‐4‐Ylmethyl)aniline **4A**

According to GPC from imine **4A′** (140 mg, 0.7 mmol) amine **4A** was obtained as light brown oil (129 mg, 91%). MS (ESI), m/z 199.120 [M + H]^+^; ^1^H NMR (300 MHz, DMSO‐d_6_) *δ* 8.47 (d, *J* = 5.8 Hz, 2H, ArH), 7.32 (d, *J* = 5.4 Hz, 2H, ArH), 6.85 (d, *J* = 8.1 Hz, 2H, ArH), 6.44 (d, J = 8.3 Hz, 2H, ArH), 6.14 (t, *J* = 6.1 Hz, 1H, NH), 4.27 (d, *J* = 6.2 Hz, 2H, CH_2_), 2.11 (s, 3H, CH_3_).

##### Chemistry: 4‐Methyl‐N‐(thiophen‐2‐Ylmethyl)aniline **6A**

According to GPC from imine **6A′** (109 mg, 0.54 mmol) amine **6A** was obtained as dark brown oil (96 mg, 87%). MS (ESI), m/z 203.870 [M + H]^+^; ^1^H NMR (300 MHz, DMSO‐d_6_) *δ* 7.34 (d, J = 4.9 Hz, 1H, ArH), 7.01 (bs, 1H, ArH), 6.98–6.91 (m, 1H, ArH), 6.87 (d, J = 8.1 Hz, 2H, ArH), 6.53 (d, *J* = 8.1 Hz, 2H, ArH), 5.99 (t, J = 5.8 Hz, 1H, NH), 4.39 (d, *J* = 5.8 Hz, 2H, CH_2_), 2.13 (s, 3H, CH_3_).

##### Chemistry: N‐Cinnamyl‐4‐Methylaniline **7A**

According to GPC from imine **7A′** (127 mg, 0.57 mmol) amine **7A** was afforded as yellow solid (121.6 mg, 95%). ^1^H NMR (300 MHz, DMSO‐d_6_) *δ* 7.40–7.19 (m, 5H), 6.88 (d, *J* = 8.2 Hz, 2H, ArH), 6.61–6.51 (m, 3H), 6.34 (dt, *J* = 15.9, 5.4 Hz, 1H, CH), 5.64 (t, *J* = 5.4 Hz, 1H, NH), 3.81 (t, *J* = 5.4 Hz, 2H, CH_2_), 2.14 (s, 3H, CH_3_).

##### Chemistry: 4‐(2‐((4‐Methylbenzyl)amino)ethyl)benzenesulfonamide **1B**

According to GPC from imine **1B′** (100 mg, 0.33 mmol) amine **1B** was obtained as white solid (73.2 mg, 73%). MS (ESI), m/z 305.131 [M + H]^+^; HRMS (ESI) calcd for C_16_H_21_O_2_N_2_S [M + H]^+^ 305.1318, found 305.1312.^1^H NMR (300 MHz, DMSO‐d_6_) *δ* 7.71 (d, *J* = 8.3 Hz, 2H, ArH), 7.38 (d, *J* = 8.3 Hz, 2H, ArH), 7.25 (s, 2H, NH_2_), 7.17 (d, *J* = 7.9 Hz, 2H, ArH), 7.09 (d, *J* = 7.9 Hz, 2H, ArH), 3.65 (s, 2H, N‐CH_2_), 2.78 −2.72 (m, 4H, CH_*2*_‐ CH_2_‐N), 2.26 (s, 3H, CH_*3*_).

##### Chemistry: 4‐(2‐((4‐Methoxybenzyl)amino)ethyl)benzenesulfonamide **2B**

According to GPC from imine **2B′** (70 mg, 0.22 mmol) amine **2B** was obtained as white solid (69.9 mg, 99%). MS (ESI), m/z 321.126 [M + H]^+^; HRMS (ESI) calcd for C_16_H_21_O_3_N_2_S [M + H]^+^ 321.1267, found 321,1264; ^1^H NMR (300 MHz, DMSO‐d_6_) *δ* 7.71 (d, *J* = 8.3 Hz, 2H, ArH), 7.38 (d, *J* = 8.2 Hz, 2H, ArH), 7.32–7.15 (m, 4H, NH_2_, ArH), 6.85 (d, *J* = 8.6 Hz, 2H, ArH), 3.72 (s, 3H, OCH_3_), 3.63 (s, 2H, N‐CH_2_), 2.78–2.71 (m, 4H, CH_2_‐CH_**2**_‐N).

##### Chemistry: 4‐(2‐(benzylamino)ethyl)benzenesulfonamide **3B**

According to GPC from imine **3B′** (100 mg, 0.35 mmol) amine **3B** was obtained as white solid (44.8 mg, 44.5%). MS (ESI), m/z 291.140 [M + H]^+^, 313.110 [M + Na]^+^; ^1^H NMR (300 MHz, DMSO‐d_6_) *δ* 7.72 (d, *J* = 8.3 Hz, 2H, ArH), 7.39 (d, *J* = 8.3 Hz, 2H, ArH), 7.28 (t, *J* = 6.5 Hz, 7H, NH_*2*_, ArH), 3.70 (s, 2H, N‐CH_2_), 2.79–2.73 (m, 4H, CH_2_‐CH_*2*_‐N).

##### Chemistry: 4‐(2‐((pyridin‐4‐Ylmethyl)amino)ethyl)benzenesulfonamide **4B**

According to GPC from imine **4B′** (100 mg, 0.35 mmol) amine **4B** was obtained as white solid (75 mg, 74.5%). MS (ESI), m/z 292.111 [M + H]^+^; HRMS (ESI) calcd for C_14_H_18_O_2_N_3_S [M + H]^+^ 292.1114, found 292.1109; ^1^HNMR (300 MHz, DMSO‐d_6_) *δ* 8.46 (d, *J* = 5.5 Hz, 2H, ArH), 7.72 (d, *J* = 8.1 Hz, 2H, ArH), 7.38 (d, *J* = 8.1 Hz, 2H, ArH), 7.30 (d, *J* = 5.4 Hz, 2H, ArH), 3.73 (s, 2H, N‐CH_2_), 2.80–2.73 (m, 4H, CH_2_‐CH_2_‐N).

##### Chemistry: 4‐(2‐((pyridin‐3‐Ylmethyl)amino)ethyl)benzenesulfonamide **5B**

According to GPC from imine **5B′** (70 mg, 0.24 mmol) amine **5B** was obtained as white solid (33.7 mg, 48%). MS (ESI), m/z 292.111 [M + H]^+^; HRMS (ESI) calcd for C_14_H_18_O_2_N_3_S [M + H]^+^ 292.1114, found 292.1112; ^1^H NMR (300 MHz, DMSO‐d_6_) *δ* 8.50 (d, *J* = 1.5 Hz, 1H, ArH), 8.42 (dd, *J* = 4.7, 1.5 Hz, 1H, ArH), 7.72 (d, *J* = 8.3 Hz, 2H, ArH), 7.70–7.67 (m, 1H, ArH), 7.39 (d, *J* = 8.3 Hz, 2H, ArH), 7.32 (dd, *J* = 8.0, 4.5 Hz, 1H, ArH), 7.26 (s, 2H, NH_2_), 3.73 (s, 2H, N‐CH_2_), 2.83‐ 2.70 (m, 4H, CH_2_‐CH_2_‐N).

##### Chemistry: 4‐(2‐((thiophen‐2‐Ylmethyl)amino)ethyl)benzenesulfonamide **6B**

According to GPC from imine **6B′** (70 mg, 0.24 mmol) amine **6B** was obtained as light brown solid (56 mg, 79%). MS (ESI), m/z 297.072 [M + H]^+^; HRMS (ESI) calcd for C_13_H_17_O_2_N_2_S_2_ [M + H]^+^ 297.0726, found 297.0722; ^1^H NMR (300 MHz, DMSO‐d_6_)*δ* 7.72 (d, *J* = 8.2 Hz, 2H, ArH), 7.43–7.33 (m, 3H, ArH), 7.25 (s, 2H, NH_2_), 6.94 (d, *J* = 3.5 Hz, 2H, ArH), 3.89 (s, 2H, N‐CH_2_), 2.78 (bs, 4H, CH_2_‐CH_2_‐N).

##### Chemistry: 4‐(2‐(cinnamylamino)ethyl)benzenesulfonamide **7B**

According to GPC from imine **7B′** (70 mg, 0.22 mmol) amine **7B** was obtained as light yellow solid (69.4 mg, 99.7%). MS (ESI), m/z 317.131 [M + H]^+^; HRMS (ESI) calcd for C_17_H_21_O_2_N_2_S [M + H]^+^ 317.1318, found 317.1315; ^1^H NMR (300 MHz, DMSO‐d_6_) *δ* 7.73 (d, *J* = 8.3 Hz, 2H, ArH), 7.45–7.16 (m, 9H, ArH, NH_2_), 6.49 (d, *J* = 16.0 Hz, 1H, CH), 6.29 (dt, *J* = 16.0, 5.9 Hz, 1H, CH), 3.32 (d, *J* = 5.9 Hz 2H, N‐CH_2_), 2.79 (bs, 4H, CH_2_‐CH_2_‐N).

## Supporting Information

**Table S1**. Glide gscore and Emodel energy contributions of the best binding conformations for each compound, including DEET; **Table S2**: Glide gscore and Emodel energy contributions of the best binding conformations for each compound, including Icaridin; **Table S3**: Representative predicted pharmacokinetic descriptors of examined compounds (the range of values corresponds to 95% of known drugs); **Figure S1**. Relative Fluorescence Intensity obtained in the absence (1‐NPN bar) and in the presence of 5 μM DEET, Icaridin, and the amines **2A, 3A, 4A, 6A, 1B, 2B** and **5B**. The observed fluorescence intensity of a solution containing 5 μM AgamOBP1 and 10 μM 1‐NPN corresponds to 100% of Intensity. The intensities of the solutions containing 5 μM AgamOBP1, 10 μM 1‐NPN, and 5 μM of ligand are shown normalized to maximum intensity; and **Figure S2**. Comparative repellent activity of amines **2A, 3A, 4A** and **6A** at the tested doses of 0.2 and 0.4 μL cm^−2^. DEET was used as a reference repellent at doses of 0.02 and 0.04 μL cm^−2^.

## Conflict of Interest

The authors declare no conflict of interest.

## Author Contributions

**Evanthia Chazapi**: investigation (equal), methodology (equal), and writing—original draft (equal). **Eftichia Kritsi**: investigation (equal), methodology (equal), writing—original draft (equal), and writing—review and editing (equal). **Constantinos Potamitis**: investigation (equal) and methodology (equal). **Panagiota G. V. Liggri**: investigation (supporting), methodology (supporting), and writing—original draft (supporting). **Katerina E. Tsitsanou**: formal analysis (equal), investigation (equal), methodology (equal), supervision (equal), writing—original draft (equal), and writing—review and editing (equal). **Christina E. Drakou**: investigation (supporting) and methodology (supporting). **Antonios Michaelakis**: formal analysis (equal), investigation (equal), methodology (equal), supervision (equal), writing—original draft (equal), and writing—review and editing (equal). **Dimitrios P. Papachristos**: investigation (supporting) and methodology (supporting). **Spyros E. Zographos**: conceptualization (equal), formal analysis (equal), investigation (equal), methodology (equal), project administration (equal), resources (equal), supervision (equal), writing—original draft (equal), and writing—review and editing (equal). **Maria Zervou**: conceptualization (equal), formal analysis (equal), investigation (equal), methodology (equal), resources (equal), supervision (equal), validation (equal), writing—original draft (equal), and writing—review and editing (equal). **Theodora Calogeropoulou**: conceptualization (lead), investigation (equal), methodology (equal), project administration (lead), supervision (lead), writing—original draft (equal), and writing—review and editing (lead).

## Supporting information

Supplementary Material

## Data Availability

The data that support the findings of this study are available from the corresponding author upon reasonable request.
