# Supplementary material for: AgamOBP1‐Directed Discovery of Repellents to Control the Spread of Mosquito‐Borne Diseases
Source: ChemMedChem. 2025 Sep 21;20(21):e202500555. doi: 10.1002/cmdc.202500555 (PMC12597222; doi:10.1002/cmdc.202500555)
Supplement: Supplementary file 1 — Supplementary Material [file CMDC-20-e202500555-s001.pdf]

Supporting Information

AgamOBP1-directed discovery of repellents to control the spread of mosquito-borne diseases

Evanthia Chazapi,[a] Eftichia Kritsi,[a],[+] Constantinos Potamitis,[a],[++], Panagiota G.V. Liggri, [a] Katerina E. Tsitsanou,[a] Christina E. Drakou,[a],[+++] Antonios Michaelakis,[b] Dimitrios P. Papachristos,[b] Spyros E. Zographos,*[a] Maria Zervou,*[a] Theodora Calogeropoulou*[a]

[a] Institute of Chemical Biology, National Hellenic Research Foundation, 48 Vassileos Constantinou Ave., 11635 Athens, Greece

[b] Benaki Phytopathological Institute, Scientific Directorate of Entomology and Agricultural Zoology, 8 Stefanou Delta Str. 14561 Kifissia, Athens, Greece

sez@eie.gr; mzervou@eie.gr; tcalog@eie.gr

[+]Current address: Laboratory of Chemistry, Analysis & Design of Food Processes, Department of Food Science and Technology, University of West Attica, Agiou Spyridonos, 12243 Egaleo, Greece.

[++]Current address: Cloudpharm Private Company, Zosimadon 13, Aigaleo, 12243 Athens, Greece

[+++]Current address: Department of Biochemistry and Biotechnology, University of Thessaly, Biopolis, 41500 Larissa, Greece

Table S1: Glide gscore and Emodel energy contributions of the best binding conformations for each compound, including DEET for the DEET binding site (pdb ID:3N7H).

**Compounds**	**Glide gscore** (kcl·mol^{-1})	**Glide emodel** (kcal·mol^{-1})	**Compounds**	**Glide gscore** (kcal·mol^{-1})	**Glide emodel** (kcal·mol^{-1})
**DEET**	-6.76	-45.93			
**1A**	-7.18	-43.40	**1B**	-10.45	-62.35
**2A**	-8.45	-46.00	**2B**	-10.03	-65.18
**3A**	-6.96	-44.74	**3B**	-10.35	-61.03
**4A**	-9.88	-45.93	**4B**	-9.59	-59.65
**5A**	-6.18	-44.02	**5B**	-10.46	-58.04
**6A**	-6.95	-41.73	**6B**	-9.38	-58.30
**7A**	-8.60	-44.48	**7B**	-10.53	-69.42

Table S2: Glide gscore and Emodel energy contributions of the best binding conformations for each compound, including Icaridin for the sIC binding site (pdb ID:5EL2)

**Compounds**	**Glide gscore** ($kcal \cdot mol^{-1}$)	**Glide emodel** ($kcal \cdot mol^{-1}$)	**Compounds**	**Glide gscore** ($kcal \cdot mol^{-1}$)	**Glide emodel** ($kcal \cdot mol^{-1}$)
**Icaridin**	-6.26	-31.07			
**1A**	-9.90	-45.26	**1B**	-10.69	-65.44
**2A**	-8.60	-47.02	**2B**	-10.26	-69.54
**3A**	-9.43	-42.38	**3B**	-9.34	-59.95
**4A**	-9.62	-44.48	**4B**	-9.45	-63.05
**5A**	-10.34	-45.85	**5B**	-10.07	-63.57
**6A**	-9.30	-42.28	**6B**	-8.16	-58.02
**7A**	-9.34	-48.74	**7B**	-9.19	-61.25

Table S3: Representative predicted pharmacokinetic descriptors of the library final products (the range of values corresponds to 95% of known drugs).

Compounds	Number of rotatable bonds (0)-(15)	Molecular Weight	Number of HB donors (0)-(6)	Number of HB acceptors (2.0)-(20.0)	QPlogPo/w (octanol water partition coefficient) (-2.0)-(6.5)	QPlogS (aqueous solubility) (-8.0)-(-1.0)	QPlogKp (skin permeability) (-8)-(-1)
**DEET**	3	191.272	0	3	2.257	-2.647	-1.447
**Icaridin**	5	229.319	1	4.2	2.404	-3.476	-2.729
**1A**	3	211.306	1	1	4.404	-4.67	-0.390
**2A**	4	227.305	1	1.75	4.075	-4.352	-0.294
**3A**	3	197.279	1	1	3.942	-4.081	-0.19
**4A**	3	198.267	1	2.5	3.107	-3.494	-0.836
**5A**	3	198.267	1	2.5	3.106	-3.494	-0.838
**6A**	3	203.301	1	1	3.92	-3.992	-0.397
**7A**	4	223.317	1	1	4.549	-4.935	-0.129
**1B**	7	304.406	3	6	1.67	-2.908	-4.778
**2B**	8	320.406	3	6.75	1.558	-2.672	-4.685
**3B**	7	290.379	3	6	1.443	-2.388	-4.579
**4B**	7	291.367	3	7.5	0.602	-1.954	-5.231
**5B**	7	291.367	3	7.5	0.615	-1.785	-5.146
**6B**	7	296.402	3	6	1.372	-2.369	-4.814
**7B**	8	316.417	3	6	2.064	-3.078	-4.351

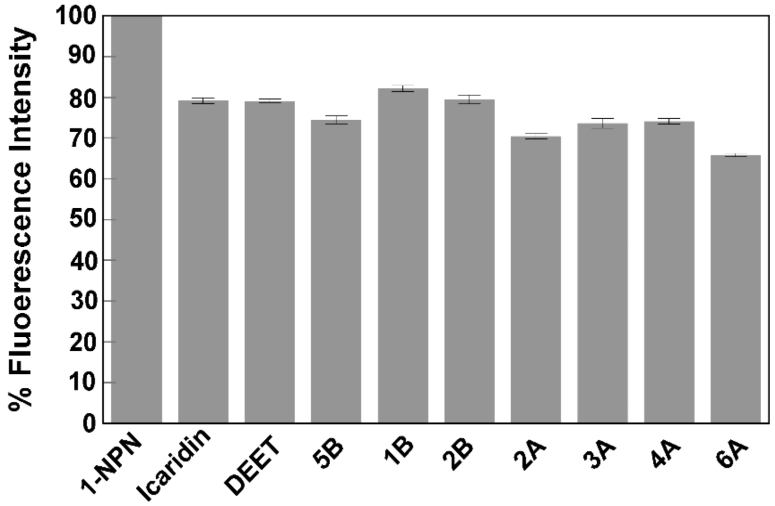

Figure S1. Relative Fluorescence Intensity obtained in the absence (1-NPN bar) and in the presence of 5 μM DEET, Icaridin, and the amines **2A, 3A, 4A, 6A, 1B, 2B** and **5B**. The observed fluorescence intensity of a solution containing 5 μM AgamOBP1 and 10 μM 1-NPN corresponds to 100% of Intensity. The intensities of the solutions containing 5 μM AgamOBP1, 10 μM 1-NPN, and 5 μM of ligand are shown normalized to maximum intensity.

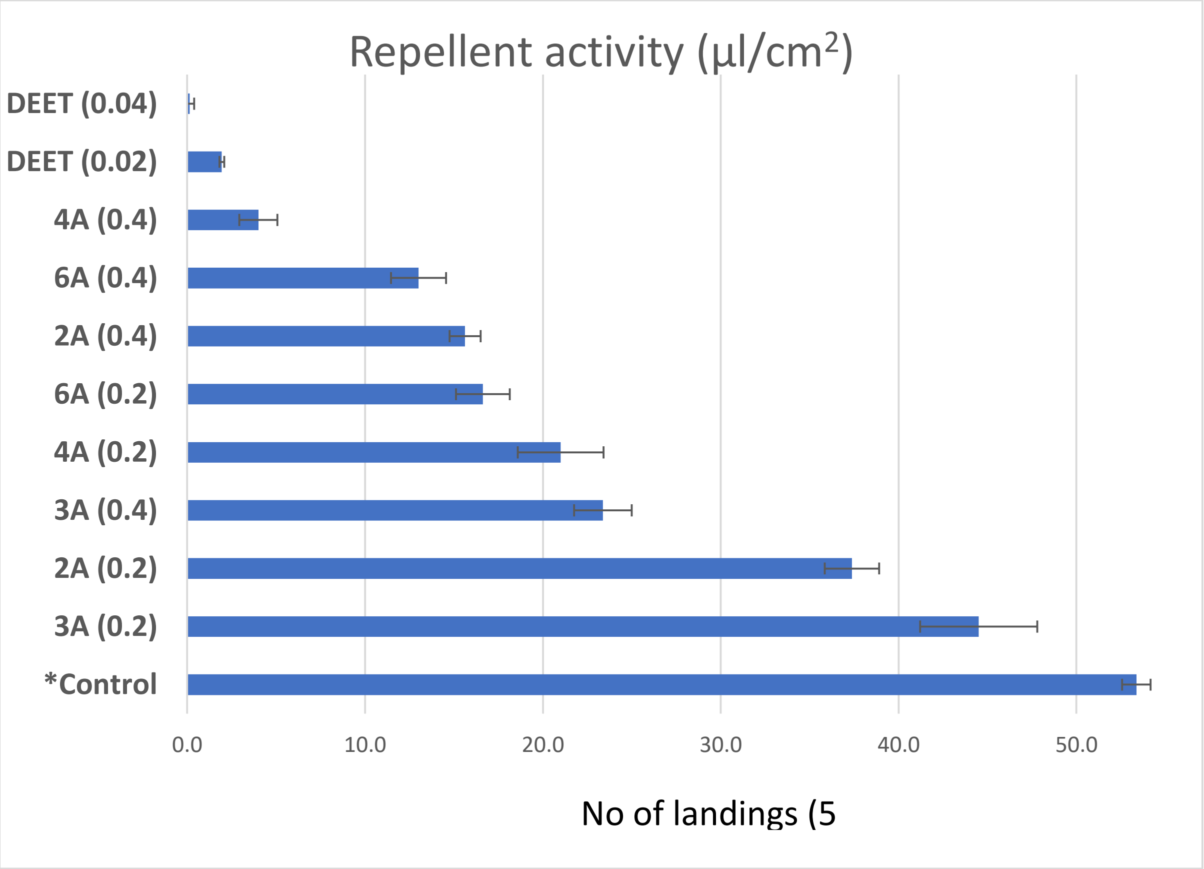

Figure S2. Comparative repellent activity of amines **2A, 3A, 4A** and **6A** at the tested doses of 0.2 and 0.4 μL/cm^2. DEET was used as a reference repellent at doses of 0.02 and 0.04 μL/cm^2.
